# Does Random Treatment Assignment Cause Harm to Research Participants?

**DOI:** 10.1371/journal.pmed.0030188

**Published:** 2006-06-06

**Authors:** Cary P Gross, Harlan M Krumholz, Gretchen Van Wye, Ezekiel J Emanuel, David Wendler

**Affiliations:** **1**Section of General Internal Medicine, Yale University School of Medicine, New Haven, Connecticut, United States of America,; **2**Section of General Internal Medicine and Yale Cancer Center, Yale University School of Medicine, New Haven, Connecticut, United States of America,; **3**Robert Wood Johnson Clinical Scholars Program, Yale School of Medicine, New Haven, Connecticut, United States of America,; **4**Section of Health Policy and Administration, Department of Epidemiology and Public Health, Yale University School of Medicine, New Haven, Connecticut, United States of America,; **5**Section of Chronic Disease Epidemiology, Department of Epidemiology and Public Health, Yale University School of Medicine, New Haven, Connecticut, United States of America,; **6**Department of Clinical Bioethics, Warren G. Magnuson Clinical Center, National Institutes of Health, Bethesda, Maryland, United States of America; University of GlasgowUnited Kingdom

## Abstract

**Background:**

Some argue that by precluding individualized treatment, randomized clinical trials (RCTs) provide substandard medical care, while others claim that participation in clinical research is associated with improved patient outcomes. However, there are few data to assess the impact of random treatment assignment on RCT participants. We therefore performed a systematic review to quantify the differences in health outcomes between randomized trial participants and eligible non-participants.

**Methods and Findings:**

Studies were identified by searching Medline, the Web of Science citation database, and manuscript references. Studies were eligible if they documented baseline characteristics and clinical outcomes of RCT participants and eligible non-participants, and allowed non-participants access to the same interventions available to trial participants. Primary study outcomes according to patient group (randomized trial participants versus eligible non-participants) were extracted from all eligible manuscripts. For 22 of the 25 studies (88%) meeting eligibility criteria, there were no significant differences in clinical outcomes between patients who received random assignment of treatment (RCT participants) and those who received individualized treatment assignment (eligible non-participants). In addition, there was no relation between random treatment assignment and clinical outcome in 15 of the 17 studies (88%) in which randomized and nonrandomized patients had similar health status at baseline.

**Conclusions:**

These findings suggest that randomized treatment assignment as part of a clinical trial does not harm research participants.

## Abstract 2

A search was conducted for RCTs where information was recorded on outcomes for participants and those who did not enter the trial, but still had access to the same treatments. No significant difference was found between randomised and non-randomised patients.

## Introduction

Despite widespread reliance on randomized clinical trials (RCTs), and claims that they represent the “gold standard” for assessing treatment efficacy, ethical concern has been raised about the impact of RCTs on participants [ 1– 4]. Specifically, there is a perception that individual patients are likely to have better outcomes when treatment decisions are based on physicians' clinical judgment, rather than random assignment [ 1, 2]. It has been claimed that by foregoing individualized treatment assignment, the process of choosing research participants' treatments by random assignment leads to an “inevitable compromise of personal care in the service of obtaining valid research results” [ 1]. Further, physician and patient concerns about random treatment assignment are among the most frequently cited reasons for refusal to enroll in RCTs [ 5– 8].

While some commentators focus on the specific impact of random treatment assignment, others have investigated the broader topic of differences in clinical outcomes between research participants and “real” patients in the community setting. Some studies have suggested that research participation may be associated with improved clinical outcomes [ 9– 14]. These data have led some to recommend trial participation as a means to better treatment [ 15]. For instance, the National Comprehensive Cancer Network's clinical practice guidelines in oncology state that “the best management for any cancer patient is in a clinical trial” [ 15]. Yet these conclusions are not based on strong evidence [ 16]. In particular, comparisons of research participants versus non-participants often include non-participants who do not meet trial eligibility criteria [ 16]. Because of stringent eligibility criteria, trial participants tend to be younger and healthier than non-participants in the community [ 16– 18]. Trial participation may also be the only means of access to some therapies: if the investigational therapy is available only in the research setting and turns out to be superior to existing therapies, trial participants who were allocated to the newer agent would be more likely to benefit. Further, the supportive clinical care that participants receive as part of research in resource-rich settings associated with some clinical trials may also be associated with superior outcomes. Recognizing these flaws in the existing data, a recent review of the literature called for more studies that assess the impact of participation in clinical research on patient outcomes in a methodologically rigorous manner [ 16].

It is particularly timely to disentangle the issues surrounding the effect of research participation on patients. There has recently been increased emphasis on designing trials that compare commercially available, clinically relevant alternatives [ 19– 21]. Some authors have advocated substantial increases in funding of “pragmatic” trials enrolling large numbers of patients in community practice settings [ 19]. Additionally, Medicare's policy has recently been modified so as to provide reimbursement for some new therapies only if patients receive them in the setting of a clinical trial [ 22]. Unlike in the previous paradigm, which viewed randomized trials as a tool to evaluate the efficacy of novel therapeutic agents, these innovations will likely result in many more patients encountering the decision of trial enrollment in the setting of routine clinical care. Prospective participants who are asked to participate in these pragmatic trials will have to decide whether to receive therapy that was selected via randomization or to select treatment with the input of their clinician.

Given the increased emphasis on recruiting large numbers of patients into trials, it is important to consider the question of enrollment from the perspective of patients who meet all eligibility criteria and are asked to enroll. If they agree to have their treatment selected at random, rather than by their clinicians or themselves, will they be more likely to experience adverse outcomes? We sought to answer this question by examining the potential risks associated with random treatment allocation, rather than delineating differences between trial participants and non-participants [ 16, 18]. While numerous studies have demonstrated the differences between trial participants and their counterparts in the community, few have focused specifically on the impact of random treatment assignment. Specifically, we were interested in the group of patients who were eligible for participation in an RCT but could also receive either of the therapies offered in the RCT even if they refused to enroll. We conducted a systematic review of published randomized controlled trials to compare the clinical outcomes of randomized patients and nonrandomized patients who were eligible for the same trial, were cared for in the same clinical setting, and received the same agents available to trial participants.

## Methods

### Selection of Studies

We conducted a Medline search to identify studies that (1) included only patients who were eligible for trial participation, (2) included only patients who were cared for at the same institutions and at the same time in which the randomized trial was recruiting, (3) allowed non-participants access to the agents used in the trial, (4) provided outcome data for both trial participants and eligible non-participants, and (5) recruited all participants in a similar manner.

The Medline search employed 23 unique combinations of terms and strings of terms (see Protocol S1). We focused a significant portion of our Medline search on identifying studies that met our definition of comprehensive cohort study design (see Protocol S1 for terms and phrases). The comprehensive cohort study design, also called the partially randomized patient preference trial design, offers eligible research participants the chance to refuse randomization but receive either the study intervention or the control intervention per study protocol [ 23]. In addition, we used the references of relevant manuscripts, authors' own bibliographic libraries, and Web of Science to identify frequently cited researchers and papers.

The Medline search identified 1,505 studies; the Web of Science search identified 371 studies. Of these 1,876 studies, the titles of 1,555 were identified by the two reviewers as potentially appropriate for inclusion in the current analysis. The abstracts of these 1,555 studies were assessed by two authors for appropriate content and relevant methodology. The full texts of 48 potentially suitable manuscripts were retrieved and assessed. Of these, 25 studies met the eligibility criteria.

### Data Analysis

An explicit abstraction instrument was used to obtain baseline characteristics of the RCT participants and eligible non-participants and primary clinical outcomes. Outcomes were restricted to the primary outcome listed in each manuscript; if more than one primary outcome was specified, the first one listed was used. To compare outcomes across studies, all study outcomes were standardized to “adverse” outcomes, e.g., for studies that reported survival, we converted probability of survival to probability of death. Most of the studies had dichotomous outcomes that enabled the calculation of odds ratios; those that did not were analyzed separately. In the two studies in which outcomes were expressed only as rates rather than as frequency counts, the stated proportion of people in each group who experienced the study outcome was multiplied by the number at baseline to estimate the frequency [ 24, 25]. In one study, non-participants were able to select from three treatment options, only two of which were part of the RCT. For this study, we included data only from non-participants who received one of the two treatments that were part of the RCT [ 25].

Because the relation between trial participation and clinical outcomes might be confounded by differences in baseline health status, we categorized the studies into three mutually exclusive groups: those in which the RCT participants were, overall, less healthy than eligible non-participants at baseline, those in which there was no clear difference in baseline health status, and those in which RCT participants were, overall, healthier at baseline. Two clinicians, using an implicit schema involving examination of baseline clinical and demographic characteristics of randomized and nonrandomized patients, independently categorized each study according to whether there was a balance of important prognostic factors between groups. Disagreements were resolved by consensus.

The odds ratios of experiencing the primary clinical outcome for RCT participants versus eligible non-participants were calculated using SAS 8.1 [ 26]. A Breslow–Day chi-square statistic indicated that it would be inappropriate to aggregate the results of studies with dichotomous outcomes because of heterogeneity. Thus, the outcomes are presented simply by study, according to baseline differences.

## Results

A total of 25 articles met the inclusion criteria and were selected for data abstraction. The dates of publication ranged from 1984 to 2002; the majority (80%) were published in 1990 or later. There was a broad range of conditions under investigation, and types of studies, including surgical trials, drug trials, and trials of counseling. The most common specialties represented were oncology (six studies), cardiovascular disease (five studies), and obstetrics/gynecology (five studies). The total number of eligible patients across all studies was 17,934 (range: 79 to 3,610); the proportion of eligible patients who agreed to be randomized ranged from 29% to 89% (average: 45 %; median: 47 %). The primary outcomes of interest varied across studies; the most common were mortality (9/25), acceptability of treatment (5/25), and proportion of time or number of days with a given condition (2/25).

### Baseline Characteristics

Table 1 shows the study intervention and enrollment data for all 25 studies, categorized according to baseline clinical and sociodemographic characteristics. There were no clear differences in baseline health status between RCT participants and eligible non-participants in 17 studies. In one study, RCT participants were healthier than eligible non-participants at baseline, and in seven studies RCT participants were less healthy at baseline than eligible non-participants. There was no significant relation between the proportion of eligible patients who agreed to be randomized and the occurrence of differences in baseline health status of randomized versus nonrandomized patients. The mean proportion of eligible patients who agreed to be randomized in the seven studies categorized as “RCT patients less healthy” was 48.9%, while the mean in the 17 studies with no baseline differences was 43.5% ( *p =* 0.61).

**Table 1:**
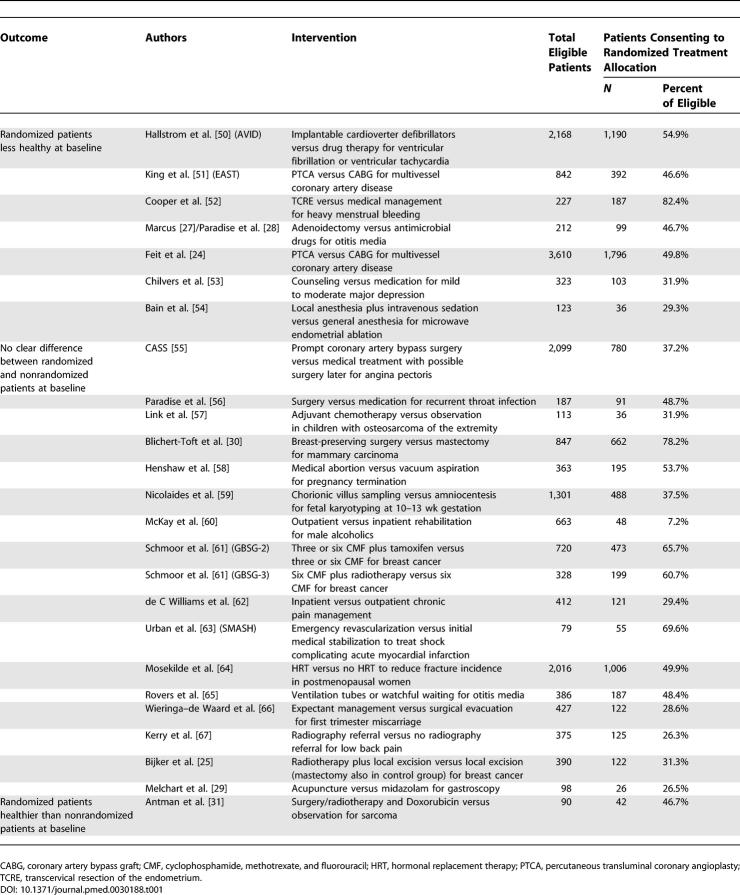
Study Characteristics

Differences in clinical sociodemographic characteristics between groups also varied in magnitude and significance. For instance, in the Bypass Angioplasty Revascularization Investigation of angioplasty versus coronary artery bypass graft, RCT participants were significantly more likely than non-participants to have a history of myocardial infarction (55% versus 51%), heart failure (9% versus 5%), or diabetes (19% versus 17%) [ 24]. Significant differences in race were found in two studies: the study by Marcus and colleagues included more non-whites in the eligible, nonrandomized group (10% versus 24%; *p* = 0.008), and the Bypass Angioplasty Revascularization Investigation included more non-whites in the RCT group (10% versus 6%, *p* < 0.001) [ 24, 27, 28].

### Outcomes

**Figure 1:**
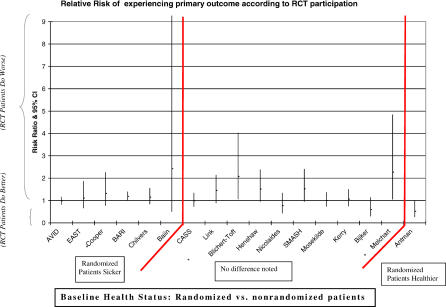
Relative Risk of Experiencing Primary Outcome According to RCT Participation Asterisks indicate statistical significance. The relevant references for the studies listed along the *x-*axis are as follows: AVID [ 50, 68], EAST [ 51], Cooper [ 52], BARI [ 24], Chilvers [ 53], Bain [ 54], CASS [ 55], Link [ 57], Blichert-Toft [ 30], Henshaw [ 58], Nicolaides [ 59], SMASH [ 63], Mosekilde [ 64], Kerry [ 67], Bijker [ 25], Melchart [ 29], and Antman [ 31].

In 22 of the 25 studies (88%), there were no significant differences in clinical outcomes between patients whose treatment was selected by randomized allocation and those whose treatment was selected on the basis of clinical judgment and/or patient preferences ( Table 2; Figure 1). There were no significant differences in clinical outcomes between randomized and nonrandomized patients in 15 of the 17 studies (88%) in which there were no clear baseline differences in health or sociodemographic status. Similarly, there were no significant differences in clinical outcomes between randomized and nonrandomized patients in six of the seven studies in which RCT participants were sicker than non-participants at baseline (86%; chi-square test, *p* > 0.05 for comparison with the “no clear baseline differences” group).

**Table 2:**
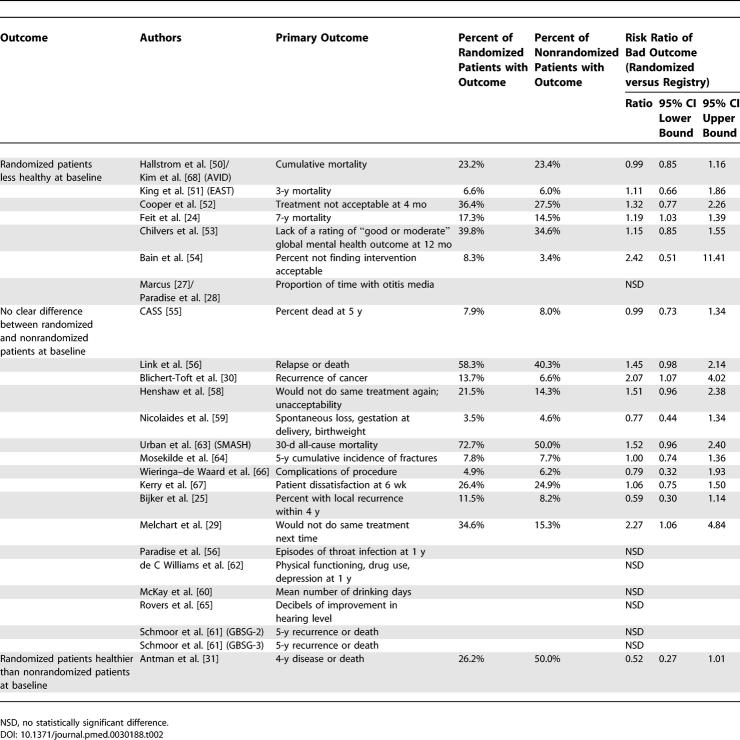
Clinical Outcome in Randomized and Nonrandomized Patients

In Feit et al.'s analysis of the data from the Bypass Angioplasty Revascularization Investigation [ 24], randomized patients were more likely to have risk factors for adverse outcomes at baseline: they were more likely to have congestive heart failure, prior myocardial infarction, or diabetes, and were more likely to be non-white and less educated. The 7-y mortality in the randomized group was 17.3%, compared with 14.5% in the nonrandomized group (relative risk: 1.19; 95% confidence interval [CI]: 1.03, 1.39) [ 24]. In Melchart et al.'s study of acupuncture versus midazolam as pretreatment for gastroscopy [ 29], there were no significant differences in baseline health status between randomized and nonrandomized groups. Randomized patients were more likely than nonrandomized patients to state that they would not undergo the same treatment again (34.6% versus 15.3%; relative risk: 2.27; 95% CI: 1.06, 4.84). Similarly, in Blichert-Toft's study of mastectomy versus breast-conserving surgery for breast cancer [ 30], randomized patients were more likely than nonrandomized patients to experience the outcome of cancer recurrence (13.7% versus 6.6%), although the difference was of borderline significance (relative risk: 2.08; 95% CI: 1.07, 4.02).

In the single study in which randomized patients were categorized as having a better baseline health status than nonrandomized patients, there was a nonsignificant trend towards the randomized patients being less likely to experience disease recurrence or death (odds ratio for randomized versus nonrandomized: 0.35; 95% CI: 0.12, 1.01) [ 31].

## Discussion

When there are several treatment options available, and there is uncertainty about which one is superior, it is assumed that individualized treatment assignment—in which clinicians consider the health status and preferences of each patient and incorporate them into a recommendation—is more likely to yield desirable outcomes. This is why doctors don't flip coins, and this is also why some may assume that randomization as part of a trial is harmful. In 23 of the 25 published clinical trials that met inclusion criteria, there were no significant differences in the likelihood of experiencing the primary study outcomes between patients whose treatment was determined by random allocation versus those whose treatment was selected on the basis of clinical judgment and/or patient preferences. More importantly, in 15 of the 17 studies in which randomized and nonrandomized patients were classified as having similar health status at baseline, there were no significant differences between these groups in clinical outcomes. These data contradict the perception that random treatment assignment as part of a clinical trial is harmful to research participants.

The finding that randomized research participants and non-participants tend to achieve similar clinical outcomes also contradicts prior studies suggesting that trial participation may be associated with superior clinical outcomes [ 9– 14]. Many of the previous studies that reported such a difference failed to account for the numerous differences between clinical care and clinical research that may influence patient outcomes, including the fact that research participants are often younger, healthier, and treated by clinicians with more experience in treating patients with the condition of interest. Specifically, we restricted the present analysis to studies that included only patients who were eligible for RCT participation and had access to similar treatments whether or not they chose to enroll in the RCT. Hence, while our study sample was therefore restricted to a relatively small subset of RCTs, our findings suggest that the purported benefit of trial participation is probably due to baseline differences between participants and non-participants, or to differences in treatments received.

All of the studies included in the present analysis allowed access to the experimental therapies to patients who refused trial enrollment. It is unclear whether our results can be generalized to randomized trials that include newer, and potentially more efficacious, therapies that are not available outside the research setting. However, a recent analysis found that only 36% of trials presented at an annual meeting of the American Society of Clinical Oncology yielded “positive” results [ 32]. These findings contradict the widespread assumption that access to experimental therapies is beneficial [ 33– 38]. Future work should explore whether participation in randomized trials of otherwise unavailable agents is associated with superior clinical outcomes.

While our comprehensive and systematic search identified far more manuscripts than prior reviews of this topic that we are aware of, our final sample size is small relative to the number of RCTs conducted annually [ 39]. As a result, although our findings were consistent across disease entities and different types of intervention, they may not be generalizable. As noted in prior reviews, many of the primary studies did not control for differences in baseline health characteristics [ 16, 39]. We used an implicit, dual review approach to account for this potential bias, stratifying manuscripts according to baseline differences between trial participants and non-participants. Ideally, future work employing primary data would enable multivariate analysis of patient-level information, to account for important patient characteristics that may affect patient outcomes. The increasing use of electronic medical records represents a tremendous opportunity for establishing longitudinal registry databases to facilitate follow-up of patients who are offered trial enrollment, yet decline.

Our results should be interpreted with several considerations in mind. We restricted our analysis to the primary outcomes assessed in the included studies. In particular, many studies assessed the outcome of mortality, and there may have been differences in the probability of other adverse events, satisfaction, or quality of life between RCT participants and non-participants. Similarly, clinical trials may include additional research procedures, such as blood draws and lumbar punctures that do not affect patient outcomes but that pose burdens to participants. Additionally, random assignment refers only to the investigational agent. Even among RCT participants, clinician-investigators generally have some latitude regarding other aspects of care that are administered to their patients and can therefore provide individualized care that consists of interventions that are distinct from the investigational agent. Similarly, clinicians may halt existing treatment for patients who are offered a choice of enrolling in a study. In these instances, if a patient is provided one of the treatment interventions offered in the study—whether selected with randomization or by patient choice—it is possible that the initial treatment may have been superior to either of the treatments under investigation. Further, publication bias might have yielded underestimates of differences between RCT participants and eligible non-participants, as investigators may have been reluctant to report data from the non-participants in their registries if they did not support the generalizability of their RCTs. Finally, there may have been important differences in health status between randomized and nonrandomized patients that were not reported by the investigators. However, given that the vast majority of the study samples in our sample found no difference in health outcome between groups, one would have to invoke a systematic over- or underestimation of health status in the randomized groups across multiple studies in order to instill bias in this synthesis.

Numerous studies indicate that RCT participants often fail to understand that their treatments will be determined by random assignment [ 18, 40– 42]. For example, a recent analysis found that half of parents who decided whether to enroll their children in a leukemia trial did not understand that treatment allocation would be determined by chance [ 18]. The failure to understand randomization is often regarded as part of a broader phenomenon, termed the “therapeutic misconception,” according to which individuals assume that research treatments are based on physicians' decisions regarding what is best for them [ 1, 43]. In this context, our findings have important implications for the informed consent process. In addition to explaining randomization, investigators should also explain that, in general, there is little evidence to support that participating in randomized trials is either helpful or harmful.

What do our findings say about the impact of clinical judgment and patient preferences on clinical outcomes? Although clinicians and patients may be reluctant to forego clinical decision-making, our data suggest that undergoing randomization, rather than individualized treatment recommendations by clinicians, is not harmful. This conclusion calls into question clinicians' ability to determine which therapy is superior for their patients in the setting of clinical equipoise, i.e., when there is uncertainty in the expert community about which treatment is superior for patients in general [ 44]. It has also been suggested that some patients who are not randomly assigned to a treatment may achieve a better outcome not because of an objective therapeutic effect, but because they were assigned to the treatment arm they preferred—a logical extension of the placebo effect [ 45]. To account for this possible “preference effect,” some have called for incorporating patient treatment preferences into the analysis phase of RCTs [ 45]. Our data provide preliminary evidence that this preference effect does not bias the outcomes of RCTs: patients who received a treatment preferred by themselves or their clinicians did not experience superior outcomes. These findings are consistent with the result of a recent review in which the authors stratified patients according to treatment received and then compared the outcome of patients who were randomized versus those who selected each therapy [ 46].

A critical barrier to enrolling patients in research studies is the fact that many patients are not even asked to participate [ 47]. One reason why physicians are reluctant to recruit their own patients is their reluctance to forego individualized treatment decisions for their patients [ 7, 48]. This reluctance is especially important because physician recommendations are among the strongest predictors of trial enrollment [ 49]. The current findings suggest that in the setting of clinical equipoise, randomized treatment allocation as part of an RCT is unlikely to be harmful This does not imply that all research is not risky, as the risks and benefits of experimental treatment may vary substantially between studies. However, in the situation in which patients will have access to the treatments that are used in the study setting regardless of whether the patient enrolls, prospective participants and their referring physicians should be reassured: there is no evidence that random treatment assignment leads to worse clinical outcomes. Furthermore, patients who do participate in such research can contribute to the important objective of improving health and well-being for all patients.

## Supporting Information

Protocol S1 Literature Search Keywords and Results (67 KB DOC)

**Editors' Summary**

**Background.**

When researchers test a new treatment, they give it to a group of patients. If the test is to be fair and provide useful results, there should also be a control group of patients who are studied in parallel. The patients in the control group receive either a different treatment, a pretend treatment (“a placebo”), or no treatment at all. But how do researchers decide who should be in the treatment group and who should be in the control group? This is an important question because the test would not be fair if, for example, all the individuals in the treatment group were elderly men and the controls were all young women, or if everyone in the treatment group received their treatment in a well-equipped specialist hospital and the controls received care in a local general hospital. Statisticians would say that the results from such studies were “confounded” by the differences between the two groups. Instead, patients should be allocated to treatment or control groups at random. Randomization also has the advantage that it can conceal from the researchers, and from the patients, whether the treatment being given is the new one or an old one or a placebo. This is important because—again for example—researchers might hold strong beliefs about the effectiveness of a new treatment and this bias in its favor might lead them, perhaps only subconsciously, to allocate younger, stronger patients to the treatment group. For these and other reasons, randomized clinical trials (RCTs) are regarded as the “gold standard” in assessing the effectiveness of treatments.

**Why Was This Study Done?**

Doctors normally decide on the “best” treatment for an individual patient based on their knowledge and experience. However, if a patient has agreed to be part of an RCT, then their treatment will instead be chosen at random. Some people worry that patients who participate in RCTs may, because their treatment is less “personalized,” have a lower chance of recovery from their illness than similar patients who are not in trials. In contrast, other argue that, particularly if the trial is part of an important research program, being in an RCT is to the patient's advantage. This study aimed to find out whether either of these possibilities is true.

**What Did the Researchers Do and Find?**

The researchers conducted a thorough electronic search of medical journals in order to find published RCTs for which information—both before and after treatment—had been recorded not only about the patients who were enrolled in the trials, but also about other patients whose condition made them eligible to participate but who were not actually enrolled. The researchers also decided in advance that they were only interested in such RCTs if the non-enrolled patients had access to the same treatment or treatments that were given to the trial participants. Only 25 RCTs were found that met these requirements. There were nearly 18,000 patients in these studies; overall 45% had received treatment after randomization and 55% had not been randomized. Most of the RCTs were for treatments for cancer, problems of the heart and circulation, and obstetric and gynecological issues. The “clinical outcomes” recorded in the trials varied and included, for example, death/survival, recurrence of cancer, and improvement of hearing. In 22 of these trials, there were no statistically significant differences in clinical outcomes between patients who received random assignment of treatment (i.e., the RCT participants) and those who received individualized treatment assignment (eligible non-participants). In one trial the randomized patients fared better, and in the remaining two the nonrandomized patients had the better outcomes.

**What Do These Findings Mean?**

These findings suggest that randomized treatment assignment as part of a clinical trial does not harm research participants, nor does there appear to be an advantage to being randomized in a trial.

**Additional Information**

**Please access these Web sites via the online version of this summary at** http://dx.doi.org/10.1371/journal.pmed.0030188.

•The James Lind Library has been created to help patients and researchers understand fair tests of treatments in health care by illustrating how fair tests have developed over the centuries

• Wikipedia, a free Internet encyclopedia that anyone can edit, has pages on RCTs

## Abbreviations

CI: confidence interval
RCT: randomized clinical trial
