# Supplementary material for: Does Random Treatment Assignment Cause Harm to Research Participants?
Source: PLoS Med. 2006 Jun 6;3(6):e188. doi: 10.1371/journal.pmed.0030188 (PMC1470665; doi:10.1371/journal.pmed.0030188)
Supplement: Supplementary file 1 [file pmed.0030188.sd001.doc]

Appendix I: Searches

| **PUBMED search terms** | **number** |
| --- | --- |
| **Searching for ANY kind of trial** |  |
| **LIMITS: in title, in English, human** |  |
| selection and bias and study | 24 |
| participation and general* | 36 |
| enroll and refus* | 19 |
| Study and partic* and baseline | 22 |
| LIMITS: all fields |  |
| generalizable and randomized | 67 |
| enroll and refus* | 19 |
| Generaliz* And enroll* and elig* | 67 |
| (Refus* AND eligibl*) AND (participat* OR enroll*) | 218 |
| Comprehensive AND cohort AND study | 729 |
| comprehensive AND cohort AND randomized | 53 |
| **LIMITS: clinical trial** |  |
| therapy and refus* and selection and bias | 7 |
| therapy and refus* and enroll | 3 |
| therapy and refus* and registry | 7 |
| enroll* and nonenroll* | 4 |
| Searching for RCTs |  |
| **LIMITS: all fields, rcts, English, human** |  |
| enroll and refus* | 2 |
| therapy and refus* and registry | 5 |
| RCT AND observational | 54 |
| RCT AND parallel | 21 |
| RCT AND concomitant | 13 |
| RCT AND registry | 13 |
| RCT AND refus* | 9 |
| randomization AND non-randomized follow-up | 14 |
| Preference (Title) and RCT | 99 |
| TOTAL | **1505** |

| **WEB OF SCIENCE search termS** | **number** |
| --- | --- |
| ARTICLE SEARCHES |  |
| Cooper KG 1997 Br J ObGyn | 10 |
| Lambert F 2000 JCE | 4 |
| Smith & Arnesen 1990 | 12 |
| AUTHOR-ARTICLE SEARCHES |  |
| **Olshewski M (author)** | 75 publications |
| Schmoor, Olschewski, Schumacher (1996) | 28 citers |
| Olschewski, Scheurlen (1985) | 19 citers |
| Olschewski, Schumacher, Davis (1992) | 13 citers |
| **Marcus SM** | 99 publications |
| Marcus SM 1990 JCE | 10 citers |
| Stat Med 2001 | 0 citers |
| Cancer 1999 | 7 citers |
| J Clin Epidemiol | 10 citers |
| **Rovers MM** | 20 publications |
| Arch Otolaryngol 2001 | 1 citer |
| Clin Otolaryngol 2001 | 0 citers |
| Arch Dis Child 2001 | 0 citers |
| Ear Hearing 2001 | 8 citers |
| Pediatrics 2000 | 8 citers |
| J Clin Epidemiol 2001 | 2 citers |
| **Albert SM** | 89 publications |
| Neurology 1997 | 18 citers |
| Davis | 43 citers |
| **Total Titles Reviewed** | **371** |
